# Pistachio Allergy: Integrating Molecular Diagnostics and Clinical Phenotypes

**DOI:** 10.3390/diagnostics16040513

**Published:** 2026-02-09

**Authors:** Julia Tworowska, Ola Sobieska-Poszwa, Agnieszka Kowalczyk

**Affiliations:** Department of Paediatrics, Allergology and Gastroenterology, Ludwik Rydygier Collegium Medicum Bydgoszcz, Nicolaus Copernicus University Torun, 85-094 Bydgoszcz, Poland; o.sobieska@gmail.com (O.S.-P.); a.kowalczyk@cm.umk.pl (A.K.)

**Keywords:** pistachio allergy, tree nut allergy, component-resolved diagnostics, molecular allergen components, basophil activation test, IgE-mediated food allergy, diagnostic algorithms, risk stratification

## Abstract

Background: Pistachio allergy is an increasingly recognized form of tree nut allergy and is strongly associated with cashew allergy due to pronounced molecular cross-reactivity. Despite its relatively low prevalence in the general population, pistachio allergy may result in severe systemic reactions and represents a significant diagnostic challenge, particularly in polysensitized patients. Objective: This narrative review aims to critically evaluate current diagnostic approaches to pistachio allergy, with a focus on molecular allergen components, mechanisms of cross-reactivity, clinical phenotypes, and the added value of advanced diagnostic tools for risk stratification. Methods: A narrative synthesis of the literature was conducted, integrating data from population-based studies, clinical cohorts, component-resolved diagnostics, basophil activation testing, and oral food challenge studies. Emphasis was placed on the diagnostic performance and clinical utility of extract-based versus molecular and functional assays. Results: Pistachio allergy is predominantly associated with sensitization to seed storage proteins, including 2S albumins, 7S vicilins, and 11S legumins, which share high sequence and structural homology with corresponding cashew allergens. This molecular relationship underlies frequent co-sensitization and clinical co-reactivity. Conventional extract-based tests show limited specificity, whereas component-resolved diagnostics and functional assays improve diagnostic precision, facilitate phenotype-based risk stratification, and may reduce the need for oral food challenges in selected patients. Conclusions: Accurate diagnosis of pistachio allergy requires an integrated approach combining clinical history with molecular and functional diagnostics. Incorporation of component-resolved diagnostics and basophil activation testing into diagnostic algorithms allows improved differentiation between asymptomatic sensitization and clinically relevant allergy, supporting individualized patient management and safer clinical decision-making.

## 1. Introduction

Tree nut allergy is a major cause of severe IgE-mediated food reactions, associated with substantial healthcare use and impaired quality of life [1]. Across Western populations, peanut/tree nut/sesame allergy affects a few percent of children and a smaller proportion of adults, with regional differences reflecting dietary exposure and diagnostic definitions [1,2]. Pistachio is an increasingly relevant trigger and, due to strong cross-reactivity with cashew, poses specific challenges for diagnosis and risk stratification [3,4,5,6].

Tree nut allergy is characterized by persistence into adulthood and a disproportionate contribution to severe and fatal food-induced reactions [2,7]. While fatalities are rare, their concentration in defined clinical phenotypes highlights the limitations of current diagnostic strategies and the need for improved risk stratification [2,7,8,9]. This unmet need is particularly evident for botanically related nuts with high rates of cross-reactivity, such as pistachio and cashew [4,8,10].

Advances in component-resolved diagnostics (CRD) and functional assays such as the basophil activation test (BAT) have improved the identification of patients at risk of clinically relevant and severe reactions [6,11]. This narrative review synthesizes current evidence on pistachio allergy, from allergen component profiles and mechanisms of cross-reactivity to diagnostic and risk stratification strategies. Particular emphasis is placed on phenotype-based clinical decision-making, including the interpretation of positive test results in the context of composite foods, the role of oral food challenges, and situations in which avoidance and education are preferable, given the low but non-negligible risk of severe reactions [6,7,12].

## 2. Methods

This narrative review was conducted using a structured literature search and expert-driven synthesis. A comprehensive search of PubMed/MEDLINE, Embase, and the Cochrane Library was performed to identify relevant publications on pistachio allergy.

The search strategy combined controlled vocabulary and free-text terms, including: “pistachio allergy”, “tree nut allergy”, “cashew cross-reactivity”, “component-resolved diagnostics”, “seed storage proteins”, “basophil activation test”, and “oral food challenge”.

Eligible publications comprised original research articles, systematic and narrative reviews, clinical cohort studies, and position papers or consensus statements addressing the epidemiology, molecular allergen characterization, mechanisms of cross-reactivity, clinical phenotypes, diagnostic strategies, and management of pistachio allergy. Case reports and conference abstracts without full peer-reviewed data were excluded unless they provided unique mechanistic insights.

Additional relevant studies were identified through manual screening of reference lists from key articles and international guidelines. Given the limited number of pistachio-specific studies, evidence related to cashew allergy and Anacardiaceae cross-reactivity was also included when directly applicable.

Data extraction focused on allergen component profiles, patterns of sensitization and clinical reactivity, diagnostic performance of extract-based and molecular tests, functional assays, and outcomes of oral food challenges and immunotherapeutic interventions. Findings were synthesized narratively, with emphasis on clinically relevant phenotypes and practical implications for diagnosis and management.

## 3. Botanical Characteristics and Taxonomic Relationships

Pistachio (*Pistacia vera* L.) belongs to the Anacardiaceae family, which also includes cashew (*Anacardium occidentale*) and mango (*Mangifera indica*), reflecting close phylogenetic relationships among these species [4]. Pistachio is a long-lived, dioecious tree adapted to dry and warm climates, producing a drupe in which the edible portion corresponds to the seed enclosed within a hard shell.

From an allergological perspective, the clinical relevance of this botanical relationship lies in the high degree of homology among seed storage proteins within the Anacardiaceae family. Pistachio and cashew share structurally and immunologically related 2S albumins and 7S/11S globulins, which constitute the molecular basis for their strong IgE cross-reactivity [4,13]. These evolutionary similarities translate into overlapping allergen component profiles and explain the frequent coexistence of sensitization and clinical allergy to both nuts.

Consistent with these molecular data, clinical and experimental studies indicate that pistachio and cashew, as sources of closely related allergens, cannot be reliably considered independent entities in diagnostic and dietary decision-making [6,7,14]. As a result, pistachio allergy is most appropriately interpreted within the broader context of Anacardiaceae-related nut allergy, with particular emphasis on cashew as a frequent primary sensitizer.

## 4. Epidemiology of Sensitization and Allergy to Pistachio

In population-based studies, tree nut allergy rarely involves a single nut, and co-sensitization and co-allergy are common. In the BAMSE cohort from Sweden, sensitization to tree nuts among 24-year-olds reached 21.2%, whereas symptoms suggestive of clinical allergy were reported by 9.8%; importantly, sensitization to seed storage proteins was more strongly associated with clinical symptoms than IgE to whole extracts [15]. These findings highlight the limited specificity of extract-based testing in the context of tree nut allergy.

Population-based epidemiological data specifically addressing pistachio are scarce. In clinical cohorts, pistachio is frequently identified as a sensitizing tree nut, which often reflects sensitization rather than confirmed clinical allergy [16]. In a Danish cohort of patients with clinically relevant tree nut allergy, nearly all individuals sensitized or reactive to cashew were also allergic to pistachio, underscoring the close clinical relationship between these two nuts [16]. Clinical cohort data further support a predominantly unidirectional pattern of reactivity from cashew to pistachio, which may be clinically useful when constructing diagnostic algorithms [16].

There are no population-based data on the prevalence of pistachio sensitization in the general population. In a study by Maloney et al., among 234 individuals with IgE-mediated peanut allergy, 76% were sensitized to pistachio, whereas clinical allergy was confirmed in only 8% of them [17]. Similarly, Andorf et al. reported pistachio sensitization in 67% of polysensitized patients with peanut allergy, illustrating that high sensitization rates do not necessarily translate into clinical reactivity [8].

In the SchoolNuts study among adolescents in Australia, the prevalence of clinically defined pistachio allergy was 1.6%, compared with 2.3% for cashew and 0.2% for sesame [18]. These data support the observation that pistachio allergy is relatively uncommon in the general population but may be overrepresented in selected clinical cohorts. In an emergency department-based study from Stockholm, pistachio was implicated in approximately 2% of food-induced allergic reactions requiring acute medical care [19].

Taken together, available data indicate that the prevalence of pistachio allergy in the general population is low, likely in the range of 1–2%, whereas in high-risk populations, particularly patients with cashew and/or peanut allergy, rates of sensitization and clinically relevant allergy are markedly higher. Importantly, across available studies, rates of pistachio sensitization consistently exceed the prevalence of clinically confirmed pistachio allergy, underscoring the need to clearly distinguish between immunological sensitization and true clinical disease when interpreting epidemiological data. This pattern reflects strong cross-reactivity with other tree nuts, especially cashew.

Regional differences in the prevalence of peanut, tree nut, and sesame allergy reflect dietary exposure, early-life feeding practices, and changing food habits [1,2,20,21]. Nuts that were previously uncommon in Western diets have gained popularity due to globalization of traditional foods and increased interest in nutrient-dense snacks [1,21]. Pistachio, once regionally consumed, is now widely eaten as a standalone snack and as an ingredient in baked goods, desserts, chocolates, and confectionery products, increasing both intentional exposure and the risk of inadvertent ingestion [1,21].

In many jurisdictions, pistachio is subject to mandatory allergen labelling, including precautionary statements related to potential cross-contamination [7,11]. Importantly, most of the pistachio allergens are largely heat-stable, and common food processing techniques have limited impact on allergenicity, which is clinically relevant in the context of composite and processed foods [12].

## 5. Pistachio Allergens

Several pistachio allergen components have been identified and characterized, most of which belong to seed storage protein families. These include Pis v 1 (2S albumin), Pis v 2 and Pis v 5 (11S legumins), Pis v 3 (7S vicilin), as well as Pis v 4, which has been described as a manganese superoxide dismutase. Together, these components account for a substantial proportion of IgE reactivity observed in pistachio-sensitized individuals [4,12,13]. An overview of pistachio allergen components and their main characteristics is provided in Table 1.

Pis v 1, a 2S albumin, is among the most frequently recognized pistachio allergen components. Similarly to other 2S albumins from tree nuts, Pis v 1 is characterized by a compact structure stabilized by disulfide bonds, conferring high resistance to heat and gastrointestinal digestion [12].

Pis v 2, Pis v 3, and Pis v 5 also contribute to pistachio allergenicity, particularly in patients with broad sensitization profiles [4,13]. These allergens share substantial sequence and structural homology with corresponding storage proteins in other tree nuts, most notably cashew, providing the molecular basis for frequent IgE cross-reactivity between these species. Pis v 4, although less extensively characterized, has been identified as an additional pistachio allergen component and may be relevant in selected patients, particularly in the context of complex or cross-reactive sensitization patterns.

From a diagnostic perspective, the identification of IgE directed against pistachio seed storage proteins, especially Pis v 1, allows for improved differentiation between clinically relevant allergy and asymptomatic sensitization compared with extract-based testing alone [6]. Nevertheless, the relative contribution of individual pistachio allergen components may vary between patients, and the clinical significance of sensitization to non–2S albumin components, should be interpreted in the broader context of co-sensitization and clinical history.

In addition to the allergen components discussed above, other putative pistachio allergens have been mentioned in the literature; however, these are based on isolated reports and remain insufficiently characterized.

## 6. Mechanisms of Sensitization and Cross-Reactivity

Sensitization to pistachio is primarily driven by IgE responses directed against seed storage proteins, a pattern characteristic of clinically relevant tree nut allergy. Within this group, 2S albumins, 7S vicilins, and 11S legumins play a central role due to their abundance, structural stability, and resistance to heat and enzymatic digestion [4,12,13]. Sensitization to these protein families is strongly associated with systemic reactions and distinguishes primary nut allergy from milder, cross-reactive phenotypes observed in pollen-associated food allergy.

The extent of cross-reactivity between different tree nuts is largely determined by botanical relatedness and sequence homology within protein families. Pistachio and cashew, both members of the Anacardiaceae family, exhibit particularly high similarity across their main seed storage proteins. For the pairs Ana o 1/Pis v 3 (7S), Ana o 2/Pis v 2 (11S), and Ana o 3/Pis v 1 (2S), sequence and structural homology of approximately 70–80% has been reported, providing a molecular basis for IgE cross-reactivity [4,13,14].

Experimental studies using IgE-binding assays and epitope mapping have demonstrated substantial overlap of IgE-binding regions between cashew and pistachio allergens, particularly within the 2S albumin family [14,22]. These findings explain the high frequency of co-sensitization observed in clinical cohorts and support the concept of cashew acting as a frequent primary sensitizer, with pistachio sensitization often arising through cross-reactive immune responses [4,10].

Clinically, this molecular relationship translates into a characteristic pattern in which sensitization or allergy to cashew is strongly predictive of reactivity to pistachio, whereas the reverse relationship is less consistent. This unidirectional pattern has been documented in multiple pediatric and adult cohorts and confirmed by oral food challenge studies, in which the majority of pistachio-allergic patients were also allergic to cashew [8,10,16]. In contrast, cross-reactivity between pistachio and more distantly related tree nuts, such as walnut or hazelnut, appears less frequent and is often mediated by broader polysensitization rather than direct botanical relatedness.

Importantly, high rates of immunological cross-reactivity do not invariably translate into clinical allergy. While IgE binding to homologous pistachio and cashew allergens is common, clinical reactivity is modulated by additional factors, including the level and affinity of specific IgE, the dominance of 2S albumin sensitization, and host-related factors such as age and comorbid atopic disease [6,7]. These considerations underscore the need to interpret sensitization patterns within a broader clinical and phenotypic context.

## 7. Clinical Manifestations of Pistachio Allergy

Pistachio allergy most commonly presents as an IgE-mediated reaction with rapid onset of symptoms, typically occurring within minutes after ingestion [4,6]. In contrast to some other tree nuts, pistachio is more frequently associated with systemic reactions rather than isolated local symptoms, reflecting the predominant role of sensitization to stable seed storage proteins [4,12,13].

Two main clinical phenotypes of pistachio allergy can be distinguished. The primary pistachio allergy phenotype is characterized by sensitization to seed storage proteins, including Pis v 1, Pis v 2, Pis v 3, and Pis v 5, and is associated with generalized clinical manifestations [6,12]. Symptoms may involve the skin (urticaria, angioedema), gastrointestinal tract (abdominal pain, vomiting), respiratory system (wheezing, bronchospasm), and cardiovascular system, with anaphylaxis reported in a subset of patients [7,8,9]. Reactions may occur after ingestion of very small amounts of pistachio, including trace exposure in composite foods such as chocolates, baked goods, or confectionery products [7,23].

A second, cross-reactive phenotype is less clearly defined for pistachio than for other nuts such as hazelnut. Unlike classic pollen–food allergy syndrome, pistachio-related reactions are rarely limited to isolated oral symptoms, and oral allergy syndrome alone should not be assumed to indicate a mild disease course [6,13]. The absence of a dominant PR-10– or profilin-driven phenotype underscores the need for cautious interpretation of positive sensitization tests and careful clinical assessment [6].

Clinical presentation is strongly influenced by coexisting nut allergies, particularly cashew allergy. Patients with confirmed cashew allergy frequently exhibit concomitant clinical reactivity to pistachio, and this combination is associated with a higher risk of systemic reactions [8,10,16]. Risk factors for severe manifestations include early age at onset, co-sensitization to 2S albumins, reactions triggered by minimal exposure, and the presence of uncontrolled asthma [6,7,8].

Population-based and emergency department-based studies indicate that tree nuts account for a substantial proportion of food-induced anaphylaxis, with pistachio implicated less frequently than cashew or walnut but still representing a clinically relevant trigger in certain regions [18,19,23,24]. Variability in reported reaction rates likely reflects differences in dietary exposure, referral patterns, and diagnostic practices.

From a clinical perspective, these findings emphasize that pistachio allergy should not be regarded as a benign or predominantly local condition. In patients with a compatible history and molecular sensitization profile, pistachio allergy represents a potentially life-threatening disease that warrants strict avoidance strategies, patient education, and access to emergency treatment [6,7,9].

## 8. Diagnosis of Pistachio Allergy

The diagnosis of pistachio allergy requires an integrated approach combining a detailed clinical history with appropriate interpretation of sensitization tests and, in selected cases, oral food challenges (OFC). The primary objective of diagnostic evaluation is to distinguish clinically relevant allergy from asymptomatic sensitization and to assess the risk of systemic reactions.

### 8.1. Clinical History

A thorough clinical history remains the cornerstone of diagnosis. Key elements include the nature and timing of symptoms following pistachio exposure, the amount ingested, and the reproducibility of reactions. Particular attention should be paid to prior tolerance of pistachio-containing products, reactions to trace exposure in composite foods, and the presence of cofactors such as physical exercise, nonsteroidal anti-inflammatory drugs, alcohol, or intercurrent illness [6,7]. A history of reactions to cashew and other tree nuts is of special importance, given the high rate of clinically relevant cross-reactivity between pistachio and cashew [8,10,16].

### 8.2. Assessment of Sensitization

Skin prick testing (SPT) and measurement of serum specific IgE (sIgE) to pistachio extract are widely used as first-line diagnostic tools. Both methods demonstrate high negative predictive value but limited specificity, particularly in polysensitized patients, due to extensive cross-reactivity among tree nuts [5,6]. A positive SPT or elevated sIgE level therefore indicates sensitization but does not reliably confirm clinical allergy.

Component-resolved diagnostics (CRD) provide improved diagnostic resolution by identifying IgE directed against individual pistachio allergen components. Sensitization to seed storage proteins, particularly Pis v 1, is more strongly associated with systemic reactions than sensitization to whole extracts or cross-reactive carbohydrate determinants [4,6,12]. Assessment of concomitant sensitization to homologous cashew components, especially Ana o 3, further refines risk stratification and may help identify patients with a high likelihood of clinical reactivity to pistachio [10,11].

Despite these advantages, validated component-specific cut-off values for pistachio allergens are currently lacking. As a result, CRD results must be interpreted in conjunction with clinical history and other diagnostic findings rather than used as stand-alone decision criteria [6].

### 8.3. Functional Testing

Functional assays, most notably the basophil activation test (BAT), assess effector cell reactivity and provide complementary information beyond IgE binding alone. In pediatric cohorts with tree nut allergy, BAT has demonstrated high diagnostic accuracy for pistachio and related nuts, with area-under-the-curve values exceeding those of conventional sensitization tests [25]. BAT may be particularly useful in polysensitized patients, in whom it can reduce the need for OFC by identifying clinically irrelevant sensitization. However, limited availability and lack of standardization currently restrict its use to specialized centers [11,25].

### 8.4. Oral Food Challenge

Oral food challenge remains the reference standard for confirming or excluding pistachio allergy. Its indication should be individualized based on clinical history and molecular sensitization profile. In patients with a convincing history of systemic reactions to pistachio, strong sensitization to seed storage proteins, or confirmed cashew allergy with Ana o 3 sensitization, OFC is often unnecessary and potentially hazardous [5,7].

Conversely, OFC may be considered in sensitized individuals with no history of pistachio ingestion or with equivocal symptoms, particularly in the absence of seed storage protein sensitization and cashew allergy. In such cases, OFC can prevent unnecessary dietary restrictions and improve quality of life [5,6]. When performed, OFC should be conducted in a specialized setting using standardized protocols with incremental dosing and appropriate emergency preparedness [5].

## 9. Proposed Diagnostic Approach for Pistachio Allergy

Given the high prevalence of co-sensitization and the limited specificity of individual tests, no single diagnostic modality is sufficient to establish or exclude pistachio allergy. A phenotype-based approach integrating clinical history, SPT/sIgE, CRD, and, when available, BAT provides the most reliable framework for diagnostic decision-making. This integrated strategy allows clinicians to balance diagnostic certainty against patient safety and to tailor management accordingly [6,7].

The diagnostic evaluation of suspected pistachio allergy should follow a stepwise, phenotype-based approach that integrates clinical history with targeted use of sensitization and functional tests. Given the high prevalence of cross-sensitization and the limited specificity of extract-based assays, the primary goal is to identify patients with clinically relevant allergy while minimizing unnecessary dietary restrictions and exposure to oral food challenges.

Initial assessment should focus on a detailed clinical history, including the nature and timing of symptoms following pistachio exposure, the amount ingested, and the presence of cofactors. Particular emphasis should be placed on prior tolerance of pistachio-containing products and on documented reactions to cashew, which strongly increase the likelihood of clinically relevant pistachio allergy [8,10,16].

In patients with a convincing history of systemic reactions to pistachio, especially in the presence of confirmed cashew allergy or sensitization to seed storage proteins, a diagnosis of pistachio allergy can often be established without further provocation. In this high-risk phenotype, additional testing serves primarily to support risk stratification rather than to confirm the diagnosis, and oral food challenge is generally not indicated due to safety concerns [5,7].

In patients without a clear history of pistachio ingestion or with equivocal or non-specific symptoms, assessment of sensitization using skin prick testing and serum specific IgE to pistachio extract represents an appropriate next step. Negative results reliably exclude clinically relevant allergy, whereas positive results require further interpretation due to frequent cross-reactivity [5,6].

Component-resolved diagnostics should be considered in sensitized patients to refine the diagnostic phenotype. Sensitization to pistachio seed storage proteins, particularly Pis v 1, and concomitant sensitization to homologous cashew components (notably Ana o 3) indicate a higher risk of systemic reactions and favour a diagnosis of true pistachio allergy [4,10,12]. In contrast, low-level sensitization limited to extract-based tests in the absence of seed storage protein sensitization suggests a lower probability of clinical reactivity.

When available, functional testing with the basophil activation test may further aid decision-making, particularly in polysensitized patients. A negative BAT supports clinical tolerance and may reduce the need for oral food challenge, whereas a positive BAT indicates effector cell reactivity and supports a diagnosis of clinically relevant allergy [11,25].

Oral food challenge should be reserved for selected low-risk patients with inconclusive diagnostic findings, no history of systemic reactions, and absence of high-risk molecular sensitization profiles. In such cases, OFC can confirm tolerance and prevent unnecessary long-term avoidance, provided it is performed in a specialized setting with appropriate safety measures [5,6].

This proposed approach emphasizes individualized risk assessment and the selective use of advanced diagnostic tools, rather than reliance on any single test. It reflects current evidence and expert interpretation and should be adapted to local resources and patient-specific factors.

## 10. Clinical Management and Immunotherapy

The cornerstone of management for confirmed pistachio allergy is strict avoidance of pistachio and pistachio-containing products, accompanied by patient and caregiver education regarding allergen labeling and the risk of inadvertent exposure. Given the frequent occurrence of reactions after trace exposure, patients should be counseled on the limitations of precautionary allergen labeling and on strategies to minimize risk in everyday settings [6,7].

Patients with a history of systemic reactions or with a high-risk sensitization profile should be prescribed an adrenaline autoinjector and receive structured training in the recognition and emergency management of anaphylaxis. Comorbid conditions, particularly asthma, should be optimally controlled to reduce the risk of severe outcomes [7,8,9].

Dietary management requires careful consideration of coexisting nut allergies, especially cashew allergy. Due to the strong clinical and molecular relationship between pistachio and cashew, avoidance strategies often include both nuts unless tolerance has been clearly demonstrated. Conversely, unnecessary exclusion of unrelated tree nuts should be avoided, as broad nut elimination can negatively impact nutrition status and quality of life [5,6].

At present, immunotherapy for pistachio allergy remains investigational. Most available data derive from studies of tree nut immunotherapy or protocols primarily targeting cashew. Notably, several studies have demonstrated cross-desensitization to pistachio during cashew oral immunotherapy, even in the absence of direct pistachio exposure, reflecting the close allergenic relationship between these nuts [26,27]. These findings suggest that cashew-targeted immunotherapy may confer secondary benefits for pistachio allergy in selected patients.

Sublingual immunotherapy has been explored mainly for pollen-associated food allergy and lipid transfer protein syndrome, but data specific to pistachio are extremely limited and insufficient to support routine use. Biologic therapies, such as omalizumab, have shown efficacy in increasing reaction thresholds in food-allergic patients and are increasingly used as adjuncts to oral immunotherapy or as monotherapy in selected settings. In this context, molecular risk stratification may, in the future, help identify selected patients with complex or high-risk sensitization profiles who could benefit from preventive biologic therapies aimed at reducing the risk of accidental reactions. While reductions in food-specific IgE levels, including pistachio, have been observed with biologic treatment, evidence for sustained tolerance following discontinuation remains limited [6,26].

**Figure 1:**
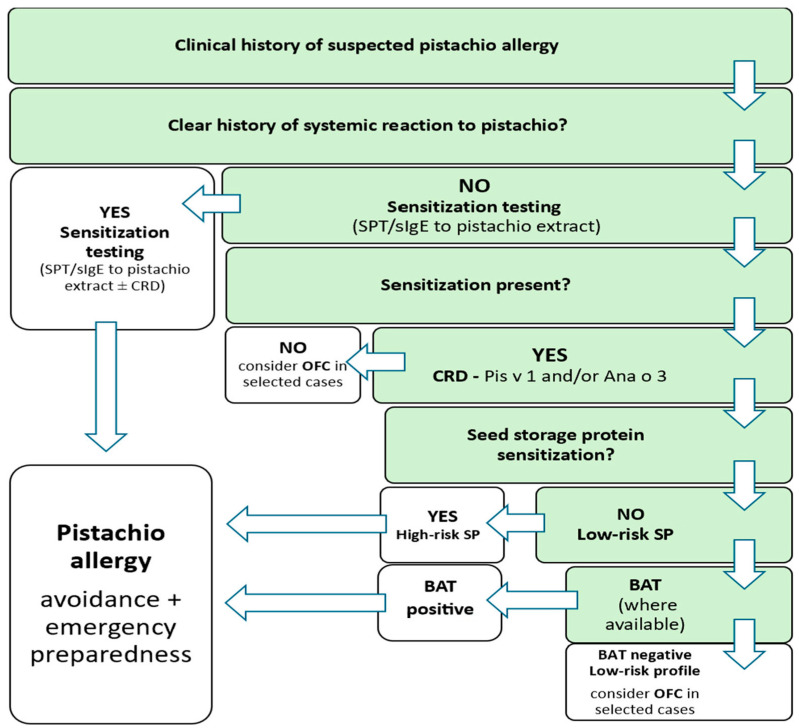
Classification tree for diagnostics and decision-making in patients with suspected pistachio allergy. High-risk sensitization profile (high-risk SP)—sIgE to seed storage proteins (Pis v 1 and/or Ana o 3). SPT—skin prick testing, sIgE—specific IgE, CRD—component-resolved diagnostics, OFC—oral food challenge, SP—sensitization profile, BAT—basophil activation test.

Overall, management of pistachio allergy should be individualized and based on a balanced assessment of clinical risk, patient preferences, and available therapeutic options. Immunotherapeutic approaches should currently be limited to specialized centers or clinical trials, pending further data on long-term efficacy and safety. The diagnostic scheme is presented in Figure 1.

## 11. Conclusions and Future Perspectives

Pistachio allergy represents a clinically relevant form of tree nut allergy that is increasingly recognized in both pediatric and adult populations. Although its prevalence in the general population remains relatively low, pistachio allergy is associated with a high risk of systemic reactions, particularly in patients with concomitant cashew allergy and sensitization to seed storage proteins.

Advances in molecular diagnostics have substantially improved the understanding of pistachio allergenicity and the mechanisms underlying cashew–pistachio cross-reactivity. Identification of IgE directed against pistachio seed storage proteins, especially Pis v 1, and assessment of homologous cashew components allow for more refined risk stratification compared with extract-based testing alone. Functional assays such as the basophil activation test further enhance diagnostic precision and may reduce the need for oral food challenges in selected patients [6,12,25].

From a clinical perspective, pistachio allergy should not be regarded as a benign or predominantly local condition. A phenotype-based diagnostic approach integrating clinical history, component-resolved diagnostics, and functional testing supports individualized decision-making and safer management strategies.

In this broader context, molecular risk stratification may, in the future, extend beyond diagnostic decision-making and help identify selected patients with complex or high-risk sensitization profiles who could benefit from preventive biologic therapies aimed at reducing the risk of accidental food-induced reactions. At present, this represents a translational perspective rather than a clinical recommendation.

Future research should focus on the validation of pistachio-specific diagnostic algorithms, including clinically meaningful component-level cut-off values and broader standardization of functional assays. In parallel, emerging data on tree nut immunotherapy, particularly cashew-targeted oral immunotherapy with cross-desensitization to pistachio, warrant further investigation in controlled studies to define long-term efficacy, safety, and optimal patient selection [26,27,28].

## Figures and Tables

**Table 1:** Pistachio Allergens and Their Clinical Relevance.

Allergen	Protein Family	Homologous Cashew Allergen	Frequency *	Key Characteristics	Clinical Relevance
Pis v 1	2S albumin	Ana o 3	Frequently reported	Small, compact protein stabilized by disulfide bonds; highly resistant to heat and gastrointestinal digestion	Key allergen component; strongly associated with primary pistachio allergy and systemic reactions
Pis v 2	11S globulin (legumin)	Ana o 2	Moderately frequent reported	Seed storage protein with high sequence homology to cashew legumin	Contributes to clinically relevant allergy, often in polysensitized patients
Pis v 3	7S globulin (vicilin)	Ana o 1	Moderately frequent reported	Abundant storage protein; structurally related to vicilins in other tree nuts	Frequently involved in cross-reactivity with cashew
Pis v 4	Manganese superoxide dismutase	Ana o 4	Rarely reported	Enzyme protein; non–seed storage protein; limited biochemical and clinical characterization	Clinical relevance unclear; described in isolated reports
Pis v 5	11S globulin (legumin)	Ana o 2	Less frequently reported	Member of the legumin family; less extensively characterized	May contribute to sensitization; clinical relevance appears variable

* Reported frequencies are based on heterogeneous study populations and assay methods; robust population-level prevalence data for individual pistachio allergen components are currently lacking.

## Data Availability

No new data were generated or analyzed in this study. Data sharing is not applicable to this article.

## References

[1] Warren C.M., Jiang J., Gupta R.S. (2020). Epidemiology and burden of food allergy. Curr. Allergy Asthma Rep..

[2] Turner P.J., Gowland M.H., Sharma V., Ierodiakonou D., Harper N., Garcez T., Pumphrey R., Boyle R.J. (2015). Increase in anaphylaxis-related hospitalizations but no increase in fatalities: An analysis of United Kingdom national anaphylaxis data, 1992–2012. J. Allergy Clin. Immunol..

[3] Patel A., Bahna S.L. (2016). Hypersensitivities to sesame and other common edible seeds. Allergy.

[4] Bastiaan-Net S., Reitsma M., Cordewener J.H.G., van der Valk J.P.M., America T.A.H.P., Dubois A.E.J., Gerth van Wijk R., Savelkoul H.F.J., de Jong N.W., Wichers H.J. (2019). IgE cross-reactivity of cashew nut allergens. Int. Arch. Allergy Immunol..

[5] Brettig T., Dang T., McWilliam  V., Peters R.L., Koplin J.J., Perrett K.P. (2021). The Accuracy of Diagnostic Testing in Determining Tree Nut Allergy: A Systematic Review. J. Allergy Clin. Immunol. Pract..

[6] Fuhrmann V., Huang H.J., Akarsu A., Shilovskiy I., Elisyutina O., Khaitov M., van Hage M., Linhart B., Focke-Tejkl M., Valenta R. (2021). From allergen molecules to molecular immunotherapy of nut allergy: A hard nut to crack. Front. Immunol..

[7] Midun E., Radulovic S., Brough H.A., Caubet J.C. (2021). Recent advances in the management of nut allergy. World Allergy Organ. J..

[8] Andorf S., Borres M.P., Block W., Tupa D., Bollyky J.B., Sampath V., Elizur A., Lidholm J., Jones J.E., Galli S.J. (2017). Association of clinical reactivity with sensitization to allergen components in multifood-allergic children. J. Allergy Clin. Immunol. Pract..

[9] Lyons S.A., Clausen M., Knulst A.C., Ballmer-Weber B.K., Fernandez-Rivas M., Barreales L., Bieli C., Dubakiene R., Fernandez-Perez C., Jedrzejczak-Czechowicz M. (2020). Prevalence of food sensitization and food allergy in children across Europe. J. Allergy Clin. Immunol. Pract..

[10] Savvatianos S., Konstantinopoulos A.P., Borgå Å., Stavroulakis G., Lidholm J., Borres M.P., Manousakis E., Papadopoulos N.G. (2015). Sensitization to cashew nut 2S albumin, Ana o 3, is highly predictive of cashew and pistachio allergy in Greek children. J. Allergy Clin. Immunol..

[11] Dramburg S., Hilger C., Santos A.F., de Las Vecillas L., Aalberse R.C., Acevedo N., Aglas L., Altmann F., Arruda K.L., Asero R. (2023). EAACI molecular allergology user’s guide 2.0. Pediatr. Allergy Immunol..

[12] Dreskin S.C., Koppelman S.J., Andorf S., Nadeau K.C., Kalra A., Braun W., Negi S.S., Chen X., Schein C.H. (2021). The importance of the 2S albumins for allergenicity and cross-reactivity of peanuts, tree nuts, and sesame seeds. J. Allergy Clin. Immunol..

[13] Scala E., Villalta D., Meneguzzi G., Giani M., Asero R. (2018). Storage molecules from tree nuts, seeds and legumes: Relationships and amino acid identity among homologue molecules. Eur. Ann. Allergy Clin. Immunol..

[14] Foo A.C.Y., Nesbit J.B., Gipson S.A.Y., DeRose E.F., Cheng H., Hurlburt B.K., Kulis M.D., Kim E.H., Dreskin S.C., Mustafa S. (2023). Structure and IgE Cross-Reactivity among Cashew, Pistachio, Walnut, and Peanut Vicilin-Buried Peptides. J. Agric. Food Chem..

[15] Bager J., Tedner S.G., Andersson N., Ballardini N., Borres M.P., Konradsen J.R., Nilsson C., Westman M., Kull I., Bergström A. (2021). Prevalence and early-life risk factors for tree nut sensitization and allergy in young adults. Clin. Exp. Allergy.

[16] Juel-Berg N., Larsen L.F., Küchen N., Norgil I., Hansen K.S., Poulsen L.K. (2022). Patterns of Clinical Reactivity in a Danish Cohort of Tree Nut Allergic Children, Adolescents, and Young Adults. Front Allergy.

[17] Maloney J.M., Rudengren M., Ahlstedt S., Bock S.A., Sampson H.A. (2008). The use of serum-specific IgE measurements for the diagnosis of peanut, tree nut, and seed allergy. J. Allergy Clin. Immunol..

[18] Sasaki M., Koplin J.J., Dharmage S.C., Field M.J., Sawyer S.M., McWilliam V., Peters R.L., Gurrin L.C., Vuillermin P.J., Douglass J. (2018). Prevalence of clinic-defined food allergy in early adolescence: The SchoolNuts study. J. Allergy Clin. Immunol..

[19] Vetander M., Helander D., Flodström C., Ostblom E., Alfvén T., Ly D.H., Hedlin G., Lilja G., Nilsson C., Wickman M. (2012). Anaphylaxis and reactions to foods in children—A population-based case study of emergency department visits. Clin. Exp. Allergy.

[20] Koplin J.J., Mills E.N., Allen K.J. (2015). Epidemiology of food allergy and food-induced anaphylaxis: Is there really a Western world epidemic?. Curr. Opin. Allergy Clin. Immunol..

[21] Pitt T.J., Becker A.B., Chan-Yeung M., Chan E.S., Watson W.T.A., Chooniedass R., Azad M.B. (2018). Reduced risk of peanut sensitization following exposure through breast-feeding and early peanut introduction. J. Allergy Clin. Immunol..

[22] He Z., Dongre P., Lyu S.C., Manohar M., Chinthrajah R.S., Galli S.J., DeKruyff R.H., Nadeau K.C., Andorf S. (2020). Identification of cross-reactive allergens in cashew- and pistachio-allergic children during oral immunotherapy. Pediatr Allergy Immunol.

[23] Ducharme L., Gabrielli S., Clarke A.E., Morris J., Gravel J., Lim R., Chan E.S., Goldman R.D., O’Keefe A., Gerdts J. (2022). Tree nut-induced anaphylaxis in Canadian emergency departments: Rate, clinical characteristics, and management. Ann. Allergy Asthma Immunol..

[24] Alves P.B., Pereira H.P., Alves M.P., Roseta L., Tavares B., Loureiro G., Carrapatoso I., Todo-Bom A., Regateiro F.S. (2022). Predictors of anaphylaxis to peanut and tree nuts in a Mediterranean population. Allergy Asthma Proc..

[25] Duan L., Celik A., Hoang J.A., Schmidthaler K., So D., Yin X., Ditlof C.M., Ponce M., Upton J.E.M., Lee J.S. (2021). Basophil activation test shows high accuracy in the diagnosis of peanut and tree nut allergy: The Markers of Nut Allergy Study. Allergy.

[26] Andorf S., Purington N., Block W.M., Long A.J., Tupa D., Brittain E., Rudman Spergel A., Desai M., Galli S.J., Nadeau K.C. (2018). Anti-IgE treatment with oral immunotherapy in multifood allergic participants: A double-blind, randomised, controlled trial. Lancet Gastroenterol. Hepatol..

[27] Elizur A., Nachshon L., Appel M.Y., Levy M.B., Epstein-Rigbi N., Koren Y., Holmqvist M., Porsch H., Lidholm J., Goldberg M.R. (2022). Cashew oral immunotherapy for desensitizing cashew–pistachio co-allergic patients. Allergy.

[28] Grabenhenrich L., Trendelenburg V., Bellach J., Yürek S., Reich A., Fiandor A., Rivero D., Sigurdardottir S., Clausen M., Papadopoulos N.G. (2020). Frequency of food allergy in school-aged children in eight European countries: The EuroPrevall-iFAAM birth cohort. Allergy.

